# A Case of Congenital Hydrocephalus

**Published:** 1849-02

**Authors:** William T. Prentis

**Affiliations:** Lewisport, Kentucky


ART. II.—*A Case of Congenital Hydrocephalus.* By WILLIAM T. PRENTIS, M.D., of Lewisport, Kentucky.

In the first number of Wood's Quarterly Retrospect, is a communication, taken from the Boston Medical and Surgical Journal, describing a case of Congenital Hydrocephalus, connected with spina bifida; varus of both feet; the labor preternatural; feet presenting. The author inquires whether cases of the kind are of very common occurrence. He wishes the question answered, and says he would like detailed circumstances and admeasurements.

Having recently had a case so very analogous to that described in the Boston Journal, I feel that it ought to be given to the medical public in detail.

Dec. 14, 1848, about 1 o'clock, A.M., I was called to see Mrs. L. in her second confinement. She had expected to be sick a month sooner. Upon my arrival she had smart, and somewhat grinding pains, with intermissions of fifteen minutes. The waters came away in great abundance, she informed me, about an hour before I saw her. After witnessing the third pain, I made an examination *per vaginam*, and touched the presenting part high up above the superior strait, and diagnosed a breech presentation—back to left acetabulum. Not seeing any obstacle in the way of a safe, if not a speedy delivery, I sat down and left the case in the hands of nature.

At 2 o'clock I touched again, and discovered a slight advancement. From this time until 10 o'clock very little progress had been made—the nates just engaging in the superior strait. I now prescribed the following:

℞. Ergot ʒj.

Aq. bullient. ℥iv.

Dose—one-third taken every fifteen minutes.

The ergot was first administered at 11 o'clock, and repeated until the whole was taken. Soon after the administration of the first dose the effect was obvious, in the increased power and duration of the pains, with shorter intervals.

At 1 o'clock, P.M., the left hip advanced under the pelvis, the right falling into the hollow of the sacrum. The pelvis, by a few powerful pains, soon emerged, and the body recovering its obliquity, advanced to about an inch above the umbilicus and stopped. The most powerful throes were of little effect. I introduced my finger in hopes of being able to bring down the arm under the pubis, but failed to find it. Traction was again commenced, and by great exertions, after a long time, the shoulders were brought down; but they remained jammed against the pubis and perineum so tightly, that the finger could scarcely be forced between them. Here the child was stationary, in spite of the powerful uterine contractions, and my assistance. After being convinced of the futility of the exertions made, in consequence of some preternatural development, either of the head or pelvis, I requested medical aid. It was now half past 2 o'clock; Dr. Stapp was sent for, and about half past 3 arrived. After making great efforts to bring the head down, but seeing nothing was accomplished, he was induced by the cries and piteous appeals of the patient, to desist. I then made similar efforts, and succeeded so far as to get my finger into the mouth; but still the head could not be made to advance, the face being to the right acetabulum, and could not be brought into the hollow of the sacrum. At this time I suggested that an attempt be made to perforate the head; but Dr. Stapp thought the operation impracticable, and I soon became convinced of the correctness of his opinion. Dr. Stapp again endeavored to deliver by bringing the face to the sacrum; but with no better success than before.

At 4 o'clock, Dr. Lewis arrived, and upon examination, and after a few efforts to extract the fœtus, concluded that the difficulty was created by a preternatural enlargement of the head, probably from hydrocephalus, an opinion in which Dr. Stapp and I concurred.

It should be remarked that I ascertained the child to be dead soon after the feet were born.

Dr. Lewis thought the child might be extracted by prolonged efforts, seeing that the pains were still very strong. At his suggestion, therefore, the woman was placed across the bed; her hips brought to the edge, with the feet supported on stools outside, in which position we had a better opportunity of affording assistance. Dr. Lewis then commenced extractive efforts, but soon gave up exhausted. I took his place, but was soon compelled to desist in consequence of my hand and fingers becoming benumbed and useless. Dr. Stapp again took the case in hand, determined, if he failed this time, to make no further attempt in that way; but being possessed of great strength, as well as skill, after sometime he succeeded in bringing the face to the sacrum, and forcing his finger under the pubis pressed upon the occiput, and, elevating the body at the same time, was fortunate enough to extract the lifeless fœtus.

I feared the consequences of such force, which appeared sufficient to tear asunder the pubal bones, and indeed take the life of the patient. The delivery was followed by considerable hemorrhage, and some indications of syncope; but by rapid friction with the hand dipped in cold water the uterus soon contracted, and the flooding was arrested. From the extrication of the shoulders to the time of the delivery of the head, was nearly four hours.

I regret that I cannot give as accurate admeasurements of the head as I could wish. I deferred taking them until next day, and then it had been taken away before my arrival. A gentleman, however, took the diameter from one parietal protuberance to the other, and found it to be seven inches. So, taking the circumference as equal to three times the diameter, it would give round the head twenty-one inches—just two and a half inches more than that of the fœtus described in the Boston Journal. I am satisfied that the average would exceed that of the Boston case.

There was no fissure of the lumbar vertebræ. The breech presented, as observed before, and there was varus of both feet.

After the patient was rendered more comfortable by the removal of the wet and bloody under-clothes, and placed properly in bed, warm fomentations to the pudenda were prescribed, and at bed time two grains of opium. She passed a tolerably good night; slept some. The next morning she complained of great general soreness, with swelling of the libia. Directed to continue warm fomentations, which after an hour were to be alternated with an application of lime-water and milk in equal quantities.

4 o'clock, P.M. The bowels not being moved I prescribed the following:

℞. Comp. ext. col. grs. x.
Extr. hyosciam. grs. iv.
M. ft. Div. in pil. iv.

Two to be taken immediately, and two at 8 o'clock if the first do not operate.

The pills were all taken, and when I visited her next day they had not operated. Gave a tablespoonful of castor oil, with directions to administer an enema at 12 o'clock, if by that time the bowels had not been moved. Her medicine operated before 12 o'clock, so that the enema was not given. Swelling of the labia diminished.

Continue fomentations, and lime water and milk. I now regard the patient as out of danger.

Dec. 18, 1848.

Notwithstanding the instructiveness of the foregoing case, we should hardly have felt ourselves justifiable in publishing it, without expressing our own decided conviction that a part of the treatment of it was wrong and not unattended with danger to the patient. To lay hold of the body of the child, for the purpose of extracting the head, in nates presentations, is never justifiable, even when the head is of ordinary size, nor is such an attempt likely to prove successful. The reason is obvious: Such tractions extend the head and bring its longest diameter into the pelvic cavity. It is only when the head is kept well flexed, with the chin upon the sternum, that it offers its smaller dimensions to the canal of the pelvis, which it has to traverse, and unless it be thus placed, no degree of force that can be applied to the body will probably succeed in bringing it through. The application of extraordinary force is not free from the risk of lacerating the organs of the patient, or contusing of them, to such a degree as may be followed by inflammation and sloughing.

If such be the difficulty of extracting the head, when of ordinary size, and so much danger be involved, we cannot think, without a shudder, of the physical strength of three male practitioners being exhausted in pulling a hydrocephalous head through the pelvis, nor dismiss our fears that the patient may not escape the consequences, which are to be dreaded. Sufficient time had not elapsed, when the case was reported, to determine its results.

We cannot agree with the gentlemen in attendance, that the head might not have been perforated to diminish its size, preparatory to its extraction by the crotchet, fixed upon such points of it as would have caused it to pass in the most advantageous manner. If the shoulders were in the way of such an operation, it would have been better to amputate them, or do anything, rather than resort to such unwarrantable force as appears to have been employed.

The history of the case would have been more satisfactory if a post-mortem examination had been made, or other measures taken more certainly to determine the cause of the preternatural size of the head. As it is, however, we thank the writer for his communication, and hope we shall receive others from him.—Ed.

